# Collagen and Alginate Hydrogels Support Chondrocytes Redifferentiation In Vitro without Supplementation of Exogenous Growth Factors

**DOI:** 10.1021/acsomega.4c01675

**Published:** 2024-05-04

**Authors:** Tosca Roncada, Gordon Blunn, Marta Roldo

**Affiliations:** † School of Pharmacy and Biomedical Sciences, University of Portsmouth, St Michael’s Building, White Swan Road, Portsmouth PO1 2DT, U.K.

## Abstract

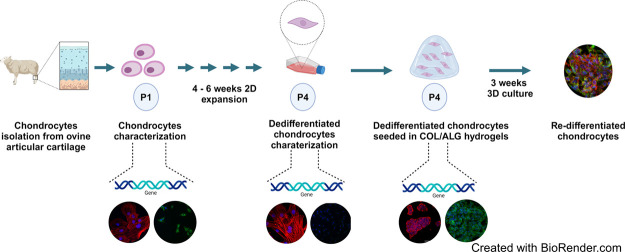

Focal cartilage defects are a prevalent knee problem affecting people of all ages. Articular cartilage (AC) possesses limited healing potential, and osteochondral defects can lead to pain and long-term complications such as osteoarthritis. Autologous chondrocyte implantation (ACI) has been a successful surgical approach for repairing osteochondral defects over the past two decades. However, a major drawback of ACI is the dedifferentiation of chondrocytes during their in vitro expansion. In this study, we isolated ovine chondrocytes and cultured them in a two-dimensional environment for ACI procedures. We hypothesized that 3D scaffolds would support the cells' redifferentiation without the need for growth factors so we encapsulated them into soft collagen and alginate (col/alg) hydrogels. Chondrocytes embedded into the hydrogels were viable and proliferated. After 7 days, they regained their original rounded morphology (aspect ratio 1.08) and started to aggregate. Gene expression studies showed an upregulation of COL2A1, FOXO3A, FOXO1, ACAN, and COL6A1 (37, 1.13, 22, 1123, and 1.08-fold change expression, respectively) as early as day one. At 21 days, chondrocytes had extensively colonized the hydrogel, forming large cell clusters. They started to replace the degrading scaffold by depositing collagen II and aggrecan, but with limited collagen type I deposition. This approach allows us to overcome the limitations of current approaches such as the dedifferentiation occurring in 2D in vitro expansion and the necrotic formation in spheroids. Further studies are warranted to assess long-term ECM deposition and integration with native cartilage. Though limitations exist, this study suggests a promising avenue for cartilage repair with col/alg hydrogel scaffolds.

## 1 Introduction

Articular cartilage (AC) is a highly specialized tissue that is essential for smooth joint movement and load transmission. Healthy AC is an avascular and aneural tissue, which is mainly composed of a proteoglycan-rich extracellular matrix (ECM), collagen type II and chondrocytes.^1^ Cartilage has limited ability to self-heal when damaged, and the current clinical cell-based therapies available include Articular Chondrocytes Implantation (ACI) and Matrix-Associated Autologous Chondrocyte Implantation (MACI). ACI is a two-step surgical procedure, in which a piece of cartilage is taken from a nonload bearing area of the damaged knee joint, from either the intercondylar notch or the superior ridge of the medial or lateral femoral condyle of the patient.^2^ Next chondrocytes are enzymatically isolated from the cartilage and expanded in vitro in monolayer for 4 to 6 weeks until a sufficient number of cells are available to be implanted into the defect site. Given that chondrocytes represent only 2% of the AC volume, the expansion procedure is essential in order to augment the limited number of isolated chondrocytes.^3,4^ However, the main disadvantage of this procedure is that chondrocytes when expanded in vitro in 2D tend to lose their original phenotype and acquire a more fibroblastic appearance.^5^ The dedifferentiation process is characterized by morphological changes as well as shifts in protein synthesis and gene expression, decreased cell proliferation, increased apoptosis, and cell senescence.^6^ Dedifferentiated chondrocytes are characterized by reduced production of cartilage-specific constituents such as collagen type II, aggrecan, and proteoglycan; while the production of nonspecific cartilage constituents such as collagen type I is increased and this leads to biomechanically inferior AC.^7,8^ Dedifferentiated chondrocytes showed reduced ability to form cartilage tissue in vivo and dedifferentiation was found to elevate the postoperative failure rate in patients after ACI.^9^ MACI is a tissue engineering strategy to repair AC that involves the use of a biopolymer membrane as a temporary scaffold to support chondrocytes adhesion, proliferation and to improve matrix deposition.^10,11^ However, MACI still fails to prevent the formation of fibrocartilage and the integration of the cells into surrounding healthy hyaline cartilage is unsatisfactory.^12^

Current strategies to prevent dedifferentiation involve culturing chondrocytes in high cell density with the addition of growth factors (e.g., TGF-β).^6,13−15^ Bianchi et al.^16^ showed that when passaged chondrocytes were cultured in vitro in high density in the presence of TGF-β3 they formed a cartilage-like tissue without acquiring a hypertrophic phenotype. However, a later in vivo study showed that using TGF-β3 treated chondrocytes did not enhance cartilage repair instead; this led to the formation of granulation tissue. Interestingly, the authors showed that when using dedifferentiated chondrocytes, not cultured with TGF-β3, the fibrocartilaginous tissue that was produced contained more collagen type II and aggrecan compared to the tissue formed by TGF-β3 treated chondrocytes.^17^ A study by Chen et al.^18^ investigated the effect of TGF-β1 on cultured human chondrocytes and revealed that genes involved in chondrocytes hypertrophy (COL10A1), blood vessel formation (Endothelial cell-specific molecule 1 (ESM1), vascular endothelial growth factor receptor 2 (KDR/VEGFR2) and vascular growth factor (VEGF)) were significantly upregulated. It is also important to consider that cells in healthy joint tissues are not subject to high levels of active TGF-β.^19,20^ On the other hand, permanent and high levels of active TGF-β were detected in OA joints.^21^ Exposing chondrocytes to high and sustained levels of TGF-β has been shown to preferentially activate the SMAD 1/5/8 pathway, which drives chondrocytes in the direction of hypertrophy. As a consequence, the effect of TGF-β on AC will lead to the expression of hypertrophic markers and the production of collagen type I^22−24^ resulting in the development of fibrocartilage rather than hyaline cartilage. As chondrocytes are the only cell type approved for cell-based therapies for AC repair, strategies to prevent or limit their dedifferentiation are required. Previous studies have provided evidence that the three-dimensional (3D) environment has a positive impact on various chondrogenic markers.^25−29^ Consequently, utilizing scaffolds and hydrogels to promote the redifferentiation of dedifferentiated chondrocytes appears to be a promising approach for preserving the chondrocyte phenotype.^30−35^

Hydrogels are hydrophilic, cross-linked polymeric networks that exhibit a unique combination of properties similar to the natural ECM, such as high water content, biodegradability and biocompatibility.^36^ Furthermore, hydrogels offer a suitable environment for cell migration, proliferation, and adhesion.^36−39^ Although numerous studies have shown successful chondrogenic induction in 3D scaffolds, very few have focused on the chondroinductivity of the hydrogels without addition of growth factors. A previous study compared the effects of alginate and hyaluronic acid hydrogels on both Wharton Jelly derived stem cells (WJ-MSC) and bone marrow derived stem cells (BM-MSC). WJ-MSC and BM-MSC exhibited distinct chondrogenic differentiation profiles at both transcript and protein levels. After 4 weeks, WJ-MSC embedded in the 3D environment were able to adapt to their surroundings and express cartilage-specific genes and matrix proteins.^40^ Our previous study showed the potential of collagen and alginate hydrogels with a stiffness of 5.75 kPa in promoting chondrogenesis in ovine mesenchymal stem cells. Remarkably, this chondrogenic differentiation was achieved without the addition of growth factors, leading to minimal collagen type I deposition.^41^ However, there is still limited information on the effects of hydrogel culture on chondrocyte redifferentiation without using growth factors. We hypothesized that collagen and alginate hydrogels with a stiffness of 5.75 kPa could serve as effective scaffolds for facilitating chondrocyte redifferentiation without the need for exogenous growth factors. In this study, chondrocytes were grown in 2D for four-6 weeks, mimicking the timeline used for ACI procedures, and allowed to dedifferentiate and embedded into collagen and alginate hydrogels to evaluate their potential for supporting chondrocytes redifferentiation.

## 2 Materials and Methods

### 2.1 Collagen Alginate (Col/Alg) Hydrogel Preparation

**Scheme 1:**
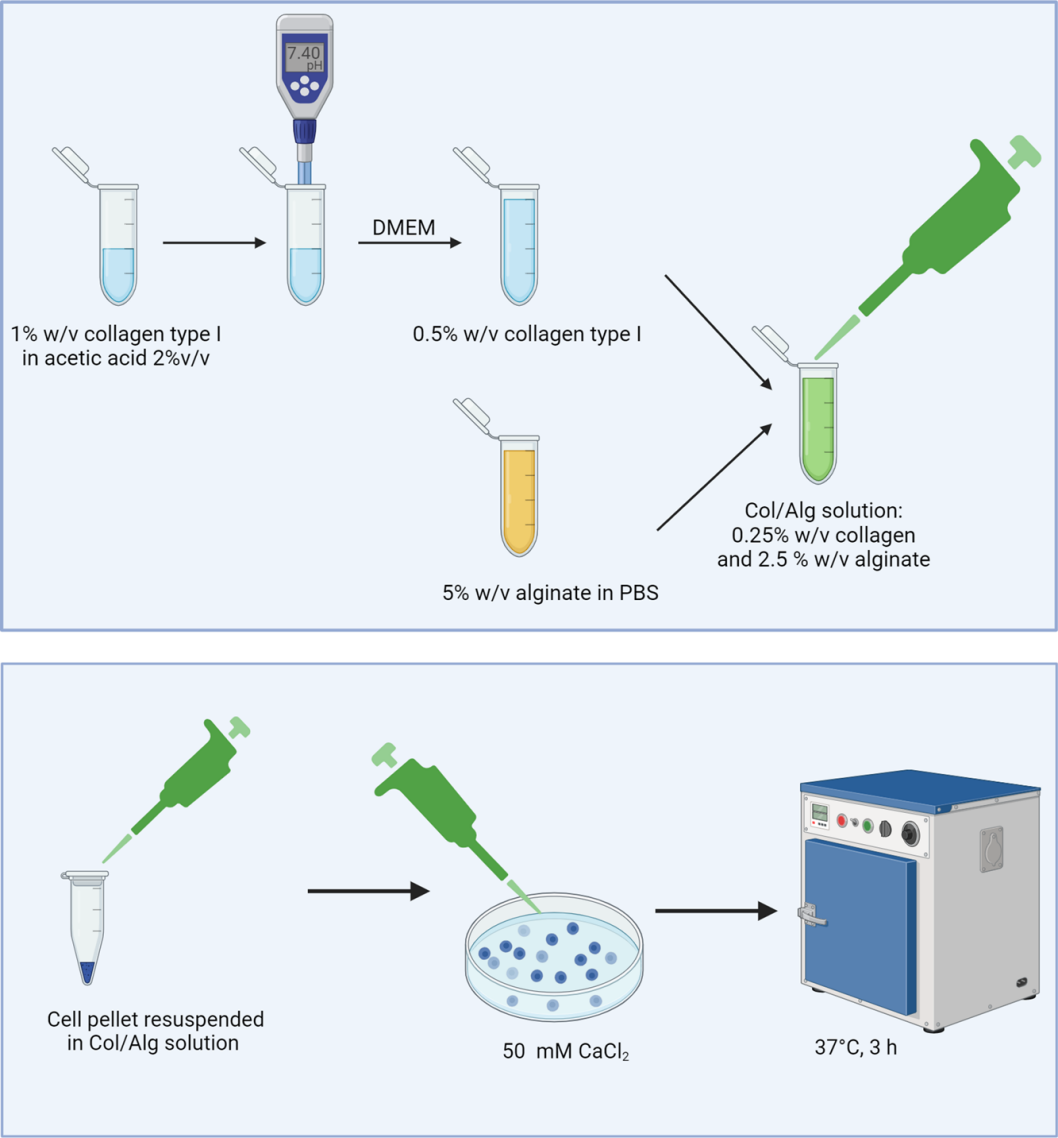
Preparation of Hydrogels and Cell Encapsulation Procedure Created with BioRender.com.

Col/alg hydrogels were prepared as previously described.^41^ Briefly, a 1% w/v collagen type I collagen solution in acetic acid (2% v/v) was prepared, and after adjusting the pH to 7.4, it was diluted with DMEM to a final concentration of 0.5% w/v. A 5% w/v alginate solution was prepared in calcium-free phosphate buffer solution (PBS 1X, pH = 7.4) (all Fisher, Loughborough, UK). The two solutions were then mixed in a 1:1 ratio and cross-linked by adding 50 μL of solution to 250 μL of 60 mM CaCl_2_. Further incubation for 3 h at 37 °C allowed complete collagen gelation (Scheme 1).

### 2.2 Ovine Chondrocytes Isolation

Primary chondrocytes were isolated from sheep AC fragments (project license number P16F4AA0A). Briefly, cartilage fragments were washed in PBS and digested overnight at 37 °C in collagenase from *Clostridium histolyticum* solution (1.5 mg/mL in DMEM/F12, supplemented with penicillin/streptomycin (1%)) under continuous agitation (all Fisher, Loughborough, UK). The next day, collagenase solution was rinsed with PBS over a 70 μm cell strainer, and the filtrate containing the cells was collected. Next, cells were harvested by centrifugation and plated (passage 0 – P0) at a density of 20,000 cells/cm^2^ in tissue culture flasks (25 cm^2^). Cells were cultured under a humidified atmosphere at 37 °C, 21% O_2_, and 5% CO_2_. After reaching confluency the cells were passaged 1:2 until P4. From P0 to P1 the time frame was about 6 to 10 days in culture depending on the donor. From passage P0 to P4 the expansion required was about 4–6 weeks. Chondrocytes at P4 were used for molecular analysis and for encapsulation procedures.

### 2.3 Hydrogel Cytocompatibility

#### 2.3.1 Cell Encapsulation

Chondrocytes at passage 4 were used for all of the experiments. All experiments were performed in triplicate, and chondrocytes isolated from AC from three different sheep were used for each experiment. Cells were incubated with trypsin-EDTA for 5 min, centrifuged, resuspended in 1 mL of DMEM supplemented with 10% (v/v) FBS (Fisher, Loughborough, UK) and counted using a hemocytometer. One million cells were pelleted by centrifugation (Eppendorf Centrifuge 5702, Eppendorf UK LTD, Stevenage, UK). The supernatant was removed and the col/alg mixture was added to the cell pellet and mixed gently to avoid bubble formation (Scheme 1). Hydrogel formation was then achieved, as described above.

#### 2.3.2 Live/Dead Staining

Ethidium homodimer-1 and calcein-AM (Fisher, Loughborough, UK) were used to evaluate the viability of chondrocytes encapsulated into the hydrogels at day 1, 7, and 14 following manufacturer’s instructions. Briefly, cells washed with Hank’s Balanced Salt Solution (HBSS) (Fisher, Loughborough, UK) were treated with 2 μM calcein-AM and 4 μM ethidium homodimer-1 for 1 h at 37 °C. Samples were rinsed two times with HBSS and imaged with a confocal microscope (LSM 880, Zeiss, Oberkochen, Germany) at 488 and 543 nm.

#### 2.3.3 Cell Proliferation

To label chondrocytes, they were detached using trypsin-EDTA (Fisher, Loughborough, UK), centrifuged, and resuspended in PBS. They were then incubated with CellTrace CFSE (Fisher, Loughborough, UK) for 20 min at 37 °C. Unconjugated CellTrace was removed by incubating the cells with DMEM/F12 supplemented with 5% FBS for 5 min at 37 °C. The cells were then centrifuged, resuspended in DMEM/F12 supplemented with 10% FBS, and analyzed.

The efficacy of cell labeling was evaluated by seeding CFSE-labeled chondrocytes (20,000 cells/cm^2^) in 24-well plates in DMEM supplemented with 10% FBS and allowing them to grow for 7 days. The remaining percentage of labeled cells was quantified on days 1, 3, and 7 by flow cytometry. CFSE-labeled chondrocytes were detected using the 488 nm laser channel (FL1). Nonstained cells were included as a control. Cells were analyzed in FACS Calibur and using CellQuest software (all BD Biosciences, Wokingham, UK).

The effect of CFSE on the metabolic activity of chondrocytes was assessed using a PrestoBlue assay (Fisher, Loughborough, UK). Cells were seeded (20,000 cells/cm^2^) in a 24-well plate in DMEM supplemented with 10% FBS and cultivated for 7 days. On days 1, 3, and 7 cells were analyzed following the manufacturer’s instructions. Nonstained cells were used as controls in each experiment.

CFSE-labeled chondrocytes embedded into col/alg hydrogels were cultivated for 1, 3, and 7 days. They were then suspended in a 0.1 M EDTA and 0.5 M sodium citrate solution at 37 °C for 10 min. Samples were then centrifuged at 300*g* for 5 min and resuspended in 500 μL of PBS. Cells were analyzed in FACS Calibur flow cytometer collecting at least 10,000 events. CFSE-labeled chondrocytes were detected using the blue 488 nm laser channel (FL1) and nonstained cells were included as a control. CellQuest software was used for the data analysis.

### 2.4 Cell Morphology Analysis

Cell morphology was assessed at P1, P4 and after embedding into col/alg hydrogels, using Phalloidin Dylight 550 (Sigma-Aldrich Company, Gillingham, UK), which selectively labels F-actin, was used. For 2D studies, chondrocytes at P1 and P4 were seeded at a density of 30,000 cells/cm^2^ on a 12 mm coverslip. After 24 h cells were washed with PBS three times and fixed with 500 μL of 4% paraformaldehyde (Sigma-Aldrich Company, Gillingham, UK) for 10 min. On day 7 hydrogels were washed with PBS three times and fixed with 500 μL of 4% paraformaldehyde for 1 h. Next chondrocytes were permeabilized with 0.2% Triton X-100 (Sigma-Aldrich Company, Gillingham, UK) in PBS for 10 min and nonspecific biding sites were blocked by incubating the cells or hydrogels in 2% BSA (Sigma-Aldrich Company, Gillingham, UK) in PBS for 30 min. Chondrocytes, both in 2D and in the hydrogels were stained with Phalloidin Dylight 550 (2 units/ml, stock solution 300 units/ml in methanol) (Fisher, Loughborough, UK) for 1 h. Nuclei counterstaining was performed by incubating cells with Invitrogen DAPI (4′,6-Diamidino-2-Phenylindole, Dihydrochloride) (Fisher, Loughborough, UK) (1:2000 dilution) for 15 min. Subsequently, chondrocytes were washed twice with PBS and imaged using confocal laser scanning microscope LSM880 at 405 and 543 nm. The aspect ratio of chondrocytes was measured using ImageJ software (National Instruments, Austin, US), for each sample (one for each donor animal) three different images in three different areas were taken and the length and width of at least 20 cells per image were measured. The aspect ratio was measured as the ratio of the length of a cell to its width.

### 2.5 RNA Extraction and RTqPCR

Hydrogels were homogenized with a pellet pestle (Thomas Fisher Scientific, USA) in 0.7 mL of qiazol (Qiagen, Switzerland) and centrifuged at 18,000*g* for 2 min at room temperature. The supernatant was transferred into a fresh Qiashredder column (Qiagen, Switzerland) and centrifuged for 2 min at 18,000*g* at room temperature. Total RNA was extracted using an RNeasy Micro Kit (Qiagen, Switzerland). RNA concentration and quality were measured using a NanoDrop (ND-1000; NanoDrop Technologies, Wilmington, DE, USA). Total RNA (250 ng from each sample) was reverse transcribed into cDNA using the High-Capacity cDNA Reverse Transcription Kit (Applied Biosystems) on a thermal cycler (Bio-Rad, Watford, UK). qRT-PCR was performed in a QuantStudio 5 Real-Time PCR System (Applied Biosystems) using SsoAdvanced Universal SYBR Green Supermix (Bio-Rad, Watford, UK) and ovine specific primers reported in Table 1 (from Eurofins Genomics, Ebersberg, Germany).

**Table 1:** Primers Employed in Gene Expression Analysis with RTqPCR

gene name	forward	reverse
GAPDH	5′-AAGGCCATCACCATCTTCCA-3′	5′-TCACGCCCATCACAAACATG-3′
SOX9	5′-TAAGGATGTGTGGAAGCCCG-3′	5′-GGGCTGAGGCAGTCTTTCAT-3′
FOXO1	5′-GCTGCAGGACAGCAAATCG-3′	5′-ATGATGTCACTGTGCGGAGG-3′
FOXO3A	5′-CTGCTGACTCCATGATCCCC-3′	5′-CTCCAGGAGCCAAGAGCC-3′
COL2A1	5′-TAAGGATGTGTGGAAGC-3′	5′-GGGCTGAGGCAGTCTTT-3′
COL1A1	5′-GAAGACCAGGGAAGCCT-3′	5′-GAAGACCAGGGAAGCCT-3′
ACAN	5′-GCTGTCTCGCCAAGTGTATG-3′	5′-ATGGTTCAGGGATGCTGACA-3′
COL10A1	5′-GCCACAAGGACCTACAGGAG-3′	5′-CAAGGAGCACAATACCCCGT-3′

### 2.6 Immunostaining and Histology

Hydrogels were fixed with 4% paraformaldehyde (Sigma-Aldrich Company, Gillingham, UK) for 1 h. They were then rinsed three times with PBS. To prevent freeze damage, the fixed gels were incubated overnight in 30% w/v sucrose (Sigma-Aldrich Company, Gillingham, UK) solution at 4 °C. The gels were then placed in optimal cutting temperature (OCT) compound (Fisher, Loughborough, UK) and frozen in isopentane (Sigma-Aldrich Company, Gillingham, UK) that had been previously chilled in liquid nitrogen. The frozen gels were stored at–80 °C for at least 1 day before sectioning. Twenty μm sections were cut with a cryostat (Leica CM3050 S Cryostat, Leica Biosystems, Milton Keynes, UK) and mounted on SuperFrost Microscope Slides (Fisher, Loughborough, UK) and were allowed to dry for 30 min. Slides were fixed in ice cold acetone (Sigma-Aldrich Company, Gillingham, UK) for 10 min at −20 °C. They were then allowed to dry for 30 min before staining. To retrieve antigens, the sections were incubated in TRIS/EDTA buffer (10 mM Tris base, 1 mM EDTA solution, 0.05% Tween 20, pH 9.0) all (Sigma-Aldrich Company, Gillingham, UK) at 95 °C for 10 min. Samples were then rinsed in PBS and incubated in a blocking buffer containing 2.5% w/v of BSA for 30 min following incubation with primary antibodies (all from Abcam, Cambridge, UK) overnight at 4 °C. Incubation with secondary antibodies for 1h at room temperature was performed after rinsing 3 times with PBS. Nuclei counterstaining was performed with DAPI (Fisher, Loughborough, UK) (1:2000 dilution, 15 min). A full list of antibodies and their dilutions is reported in Table 2. Samples were then washed three times with PBS and mounted with aqueous fluorescent mounting media and imaged using a confocal laser scanning microscope LSM880 at 405, 488, and 594 nm channels. For histology, slides were fixed with 70% ethanol and rinsed with deionized water. Slides were then stained for 90 s with filtered 0.1% Mayers Hematoxylin (Fisher Scientific, Loughborough, UK) followed by washing in tap water, 1% acetic alcohol, and 70% ethanol. Slides were then stained with eosin (3 min) followed by washes in 70% and 100% ethanol and xylene. Samples were mounted with DPX and imaged using an EVOS FL Auto 2 Cell Imaging System (Fisher Scientific, Loughborough, UK).

**Table 2:** List of Antibodies and Dilutions

	antibodies	dilution
primary antibodies	rabbit monoclonal [EPR7785] to collagen I	1:500
	anti-aggrecan antibody [6-B-4]	1:300
	anti-collagen II antibody	1:300
	recombinant anti-collagen VI antibody [EPR17072]	1:300
secondary antibodies	goat anti-rabbit IgG H&L (Alexa Fluor 594)	1:300
	sheep anti-mouse IgG (whole molecule) F(ab′)2 fragment–FITC	1:300

## 3 Results

### 3.1 Chondrocytes Characterization

**Figure 1:**
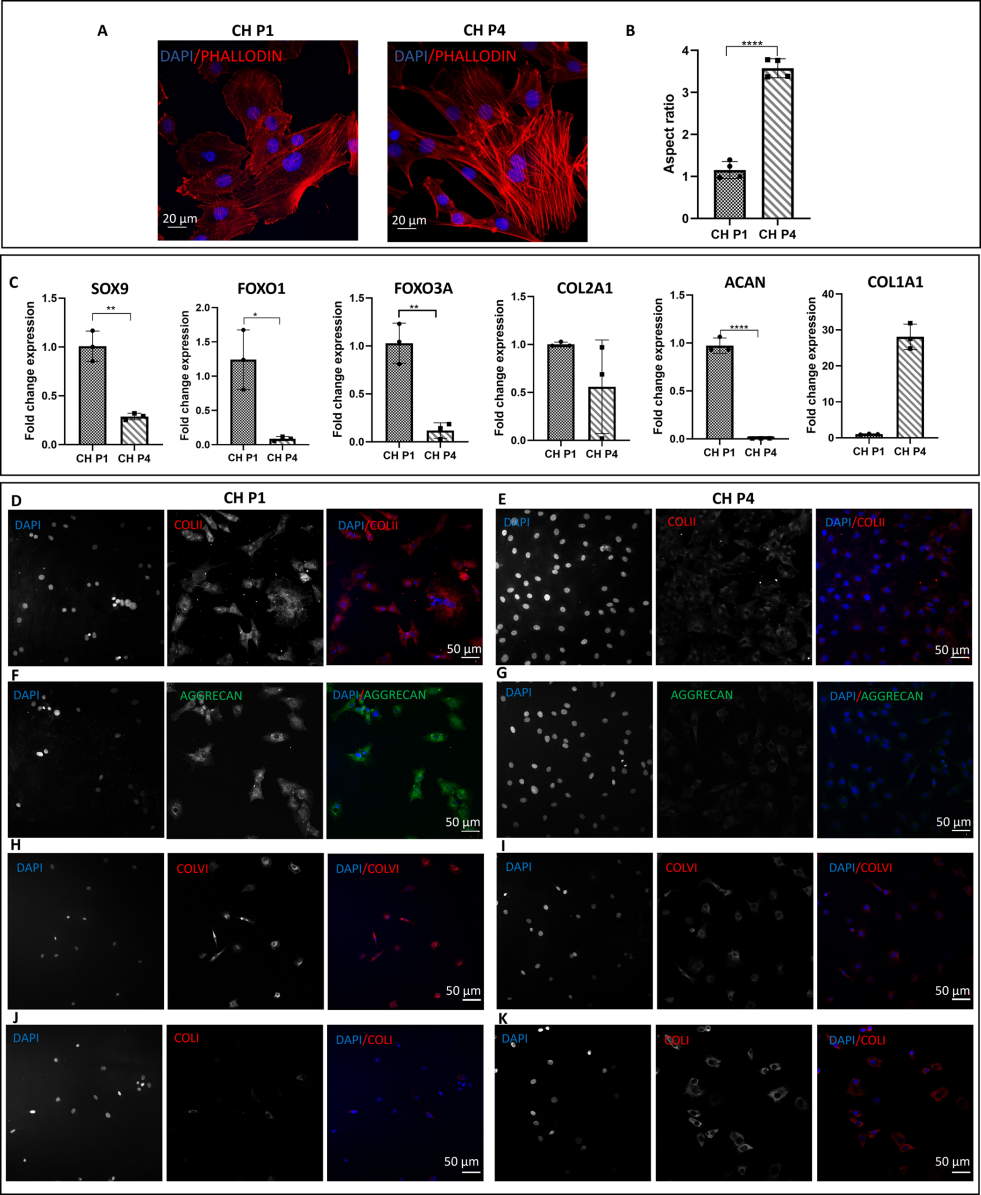
Chondrocytes (CH) characterization at P1 and P4. (A) Representative confocal images of phalloidin and DAPI staining of chondrocytes P1 and chondrocytes P4. (B) Aspect ratio analysis of chondrocytes at P1 and P4. Error bars denote standard deviation, *n* = 4. Comparison between groups was assessed by unpaired *t* test, *p* < 0.0001. (C) Expression of typical chondrogenic markers evaluated at mRNA level presented as fold change using the 2^–ΔΔCt^ method relative to chondrocytes P1. Error bars denote standard deviation, *n* = 3. Comparison between groups was assessed by unpaired *t* test. **** = *P* < 0.0001, ** *P* = 0.0055; Immunofluorescence staining of (D) collagen type II, (F) aggrecan, (H) collagen type VI, (J) collagen type I of chondrocytes at P1 and (E, G, I, K) and P4.

Chondrocytes were characterized at passages P1 and P4. The morphology of chondrocytes was assessed by using phalloidin and DAPI staining. At P1, the chondrocytes exhibited the characteristic rounded morphology (Figure 1A) and had an aspect ratio of 1.15 (Figure 1B). However, when chondrocytes were grown in a 2D environment, they lost their original morphology and at P4 acquired a spindle-shaped appearance with abundant stress fibers and an aspect ratio of 3.57 (Figure 1A,B). Molecular analysis was conducted to examine the expression of typical chondrogenic markers in different passages. At P1, the chondrocytes expressed markers such as SOX9, FOXO1, FOXO3A, ACAN, and COL2A1, indicative of their chondrogenic phenotype. However, in 2D, as passages increased, there was a significant reduction in the expression of chondrogenic markers and a notable increase in the expression of COL1A1, a marker associated with chondrocyte hypertrophy (Figure 1C). Furthermore, passaging had an impact on the deposition of the cartilage ECM with a decrease in the deposition of components such as collagen type II and aggrecan, which are essential for maintaining cartilage structure and function. Immunofluorescence staining confirmed that at P1, chondrocytes exhibited positive staining for collagen type II, aggrecan (*p* < 0.0001), and collagen type VI (Figure 1D–K). However, at P4, a reduction in the deposition of ECM proteins was observed, accompanied by an increased level of deposition of collagen type I (Figure 1D–K). This shift in matrix composition suggests a phenotypic change toward a more fibroblastic cell type.

### 3.2 Cytocompatibility of Col/Alg Hydrogels

**Figure 2:**
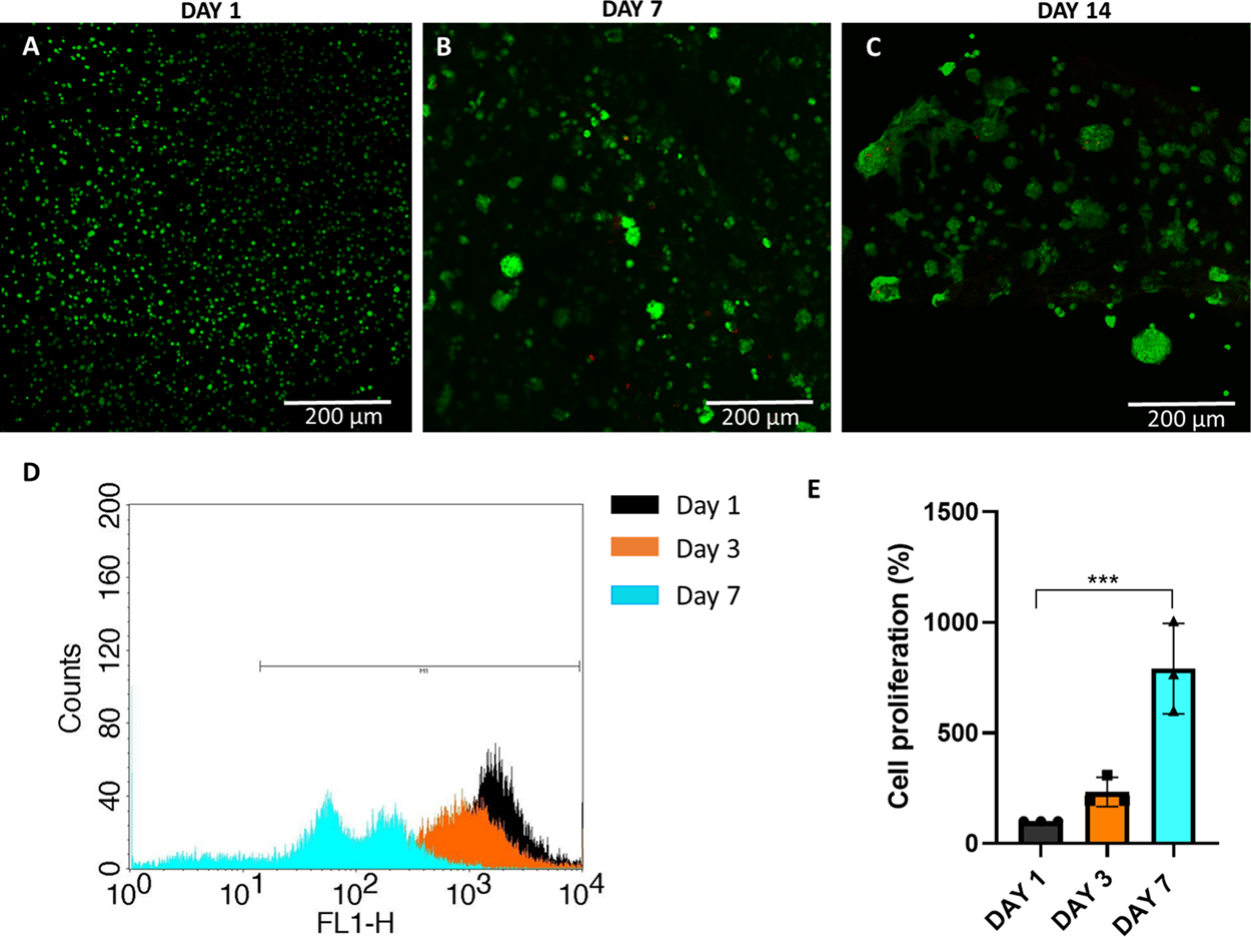
Viability and proliferation of P4 chondrocytes in col/alg hydrogels. (A–C) Representative images of live and dead staining using calcein-am and ethidium homodimer-1 to determine the viability of dedifferentiated chondrocytes embedded into col/alg hydrogels at days 1, 7, and 14. (D) Flow cytometry histogram of the frequency distribution of CFSE stained chondrocyte at day 1, 3, and 7. (E) Cell proliferation analysis of chondrocytes into col/alg hydrogels at day 1, 3, and 7. Error bars denote standard deviation, *n* = 3. Comparison between groups was assessed by ordinary one-way ANOVA using post hoc Dunnett’s test *n* = 3, * *p* = 0.05, **** *p* < 0.0001.

To evaluate the viability of chondrocytes at P4 within the collagen/alginate (col/alg) hydrogels, a live/dead assay was conducted at different time points (days 1, 7, and 14). The results, shown in Figure 2A–C, demonstrate that embedding P4 chondrocytes into the col/alg hydrogels did not have a detrimental effect on cell viability as the cells remained viable within the hydrogels for up to 14 days. The distribution of chondrocytes within the col/alg hydrogels was evaluated over time. On day 1, the chondrocytes appeared to be uniformly dispersed throughout the hydrogels. However, as the culture period progressed, distinct changes became apparent. From day 7 onward, chondrocytes started to rearrange themselves and form spherical colonies within the hydrogels. This was associated with cells migration, which overall affected cell density in some areas of the hydrogels.

Cell proliferation was assessed using CFSE Celltrace staining, where a reduction of fluorescence over time indicates the proliferation of chondrocytes. Initially, cell labeling optimization was conducted in a 2D monolayer culture using two concentrations of CellTrace dye (5 and 10 μM). The viability and proliferation of cells labeled with CellTrace were compared to the unstained control, and it was determined that the use of CellTrace did not have an adverse effect on cell viability and proliferation, even at the highest concentration tested (Figure S1). To evaluate labeling efficacy, the percentage of CFSE-positive cells was monitored for 7 days using flow cytometry. After 7 days, over 90% of cells were positive for CFSE staining; however, the difference between the two staining concentrations was significant, and 10 μM was used to assess the proliferation of dedifferentiated chondrocytes into the col/alg hydrogels. P4 chondrocytes were labeled with CFSE and then embedded into the col/alg hydrogel. The proliferation of these cells within the hydrogel was monitored over a 7-day period. Flow cytometry analysis revealed a decrease in fluorescence intensity from day 1 to day 7, indicating that the cells embedded in the hydrogel were undergoing proliferation (Figure 2D,E).

### 3.3 Morphology of P4 Chondrocytes within Col/Alg Hydrogels

**Figure 3:**
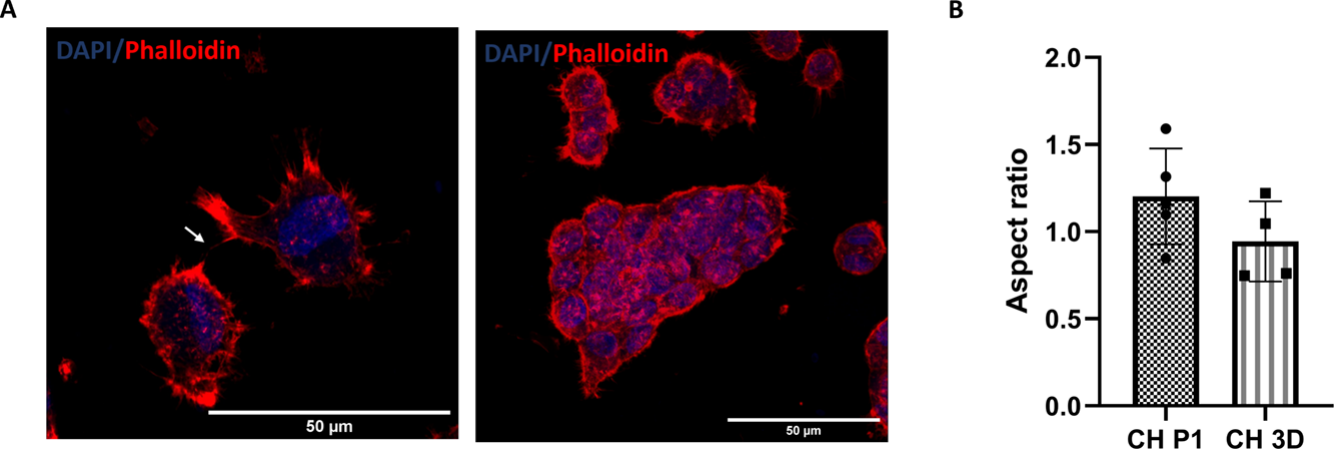
P4 chondrocytes morphology into col/alg hydrogels. (A) Morphological analysis of dedifferentiated chondrocytes embedded into hydrogels at day 7. (B) Aspect ratio analysis comparing chondrocytes at p1 and dedifferentiated chondrocytes embedded into hydrogels at day 7. Error bars denote standard deviation, *n* = 4. Comparison between groups was assessed by unpaired *t* test, *p* > 0.05.

Phalloidin and DAPI staining were used to evaluate the cellular morphology of P4 chondrocytes within the col/alg hydrogels after 7 days of culture. Figure 3 shows that the chondrocytes regained their characteristic rounded morphology and did not present abundant stress fibers when cultured within the col/alg hydrogels. Furthermore, chondrocytes started to form an interconnection and self-assembled into clusters. This observation highlights the ability of the col/alg hydrogels to support cellular interactions. The formation of interconnections and clusters suggests the initiation of cell–cell communication and the establishment of tissue-like structures, which are important aspects of chondrocyte redifferentiation and the development of functional cartilage tissue.^42^

Quantitative analysis revealed that after 7 days of culture, the chondrocytes in the col/alg hydrogels exhibited an aspect ratio of 1.08, which was not significantly different from their original aspect ratio at P1. This indicates that the chondrocytes within the hydrogels regained their characteristic rounded shape, resembling their native morphology.

### 3.4 Gene Expression of P4 Chondrocytes Embedded into Col/Alg Hydrogels

**Figure 4:**
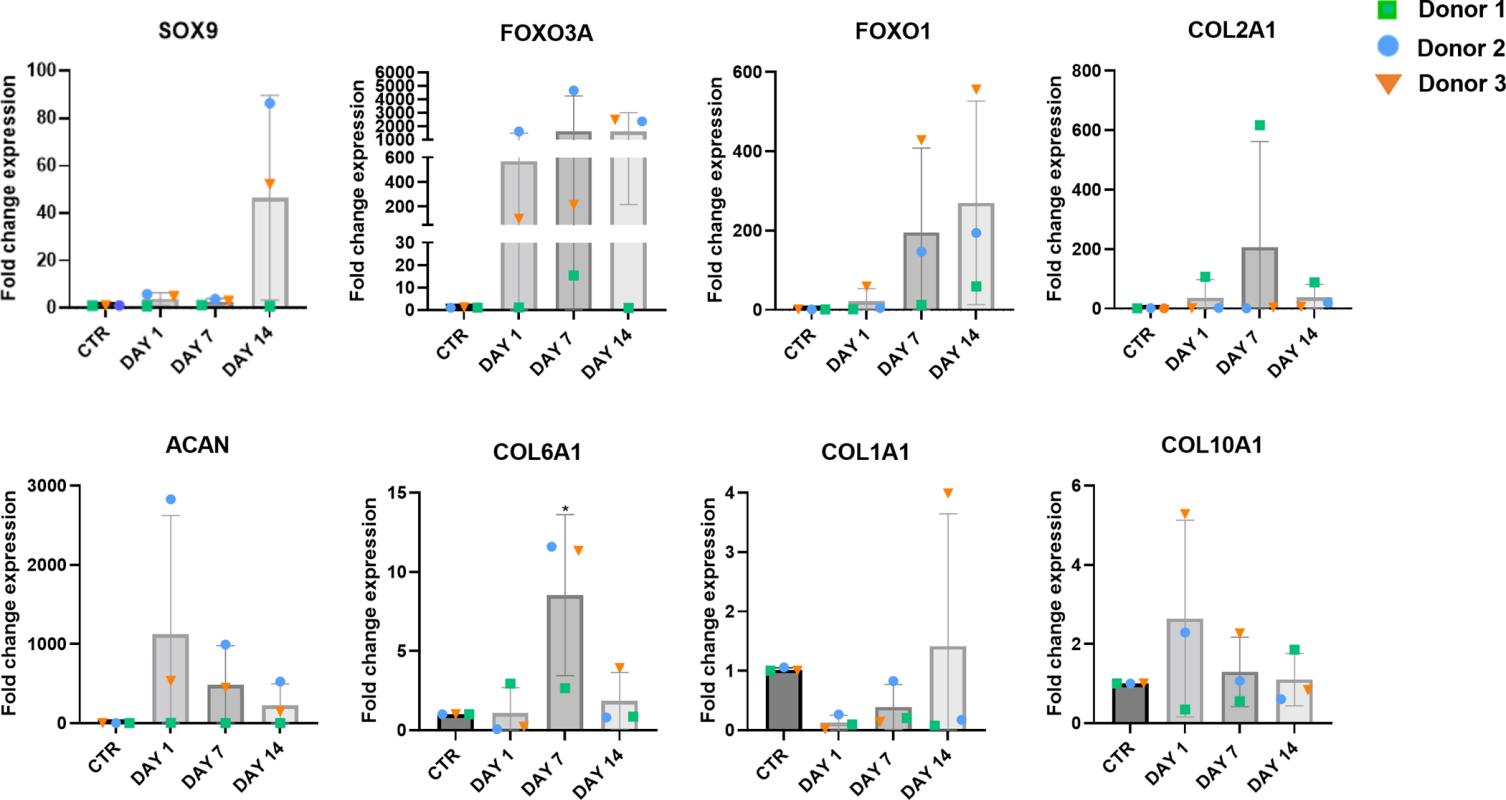
Gene expression of P4 chondrocytes embedded into col/alg hydrogels at day 1, 7, and 14. The effect of the 3D culture on dedifferentiated chondrocytes compared to dedifferentiated chondrocytes in 2D, mRNA data are presented as fold change relative to the baseline reference of cells grown in 2D before seeding onto hydrogels (time zero), this is indicated a CTR. Results represent mean ± SD (*n* = 3). Comparison between groups was assessed by ordinary one-way ANOVA using post hoc Dunnett’s test.

The gene expression analysis was performed on chondrocytes embedded into col/alg hydrogels on days 1, 7, and 14. Data for P4 chondrocytes grown in 2D were used as time zero, labeled as the control in the graphs. Chondrocytes that continued to be grown in 2D in parallel to those that were transferred to the hydrogels did not show any change in shape, gene expression, or protein production (data not shown). Although the changes in the expression of the chondrogenic markers SOX9, FOXO1, and FOXO3A were not significant when chondrocytes were cultures in 3D, a trend could be observed on day 14 (Figure 4). In addition, the 3D culture affected the expression of ECM proteins, where at day 7 a significant increase in the expression of COL6A1 was measured. Compared to time zero, an increase of COL2A1 expression was measured in chondrocytes in the hydrogel at days 7 and 14, indicating the potential of the 3D culture to promote the production of cartilage-specific ECM protein. The 3D culture influenced the expression of hypertrophic markers on day 1, and at day 7 a reduction in the expression of COL1A1 was observed. On day 1 an increase in gene expression of COL10A1 was observed; however, on days 7 and 14 the expression of COL10A1 remained comparable to the 2D control. This may suggest that the 3D culture system stabilizes the expression of COL10A1 over time, potentially preventing the further progression of chondrocyte hypertrophy.

### 3.5 Histology and Immunostaining of P4 Chondrocytes into Col/Alg Hydrogels

**Figure 5:**
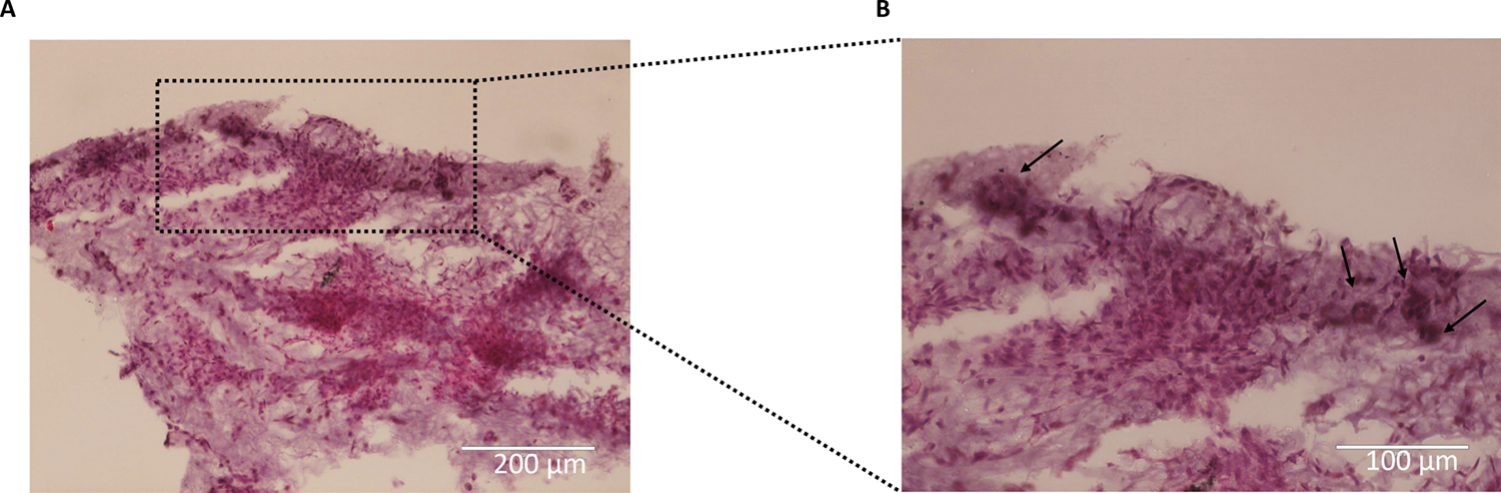
Hematoxylin and eosin (H&E) staining of P4 chondrocytes embedded into hydrogels on day 21. (A) Chondrocytes embedded into col/alg hydrogels at day 21 stained with H&E scale bar 200 μm; (B) magnification of images A, arrow indicates the presence of chondrocytes aggregates within the tissue, scale bar 100 μm.

On day 21, H&E staining of the hydrogels containing chondrocytes revealed extensive colonization of the entire hydrogel, with the formation of large spherical cell clusters (Figure 5). The presence of chondrocyte aggregates was still evident within the hydrogels, indicating their sustained growth and maturation (Figure 5). However, it was observed that the aggregates were undergoing fusion, gradually merging together to form a more tissue-like structure. This observation suggests the development of a more organized and integrated chondrocyte network within the hydrogel, indicative of tissue formation and maturation.

**Figure 6:**
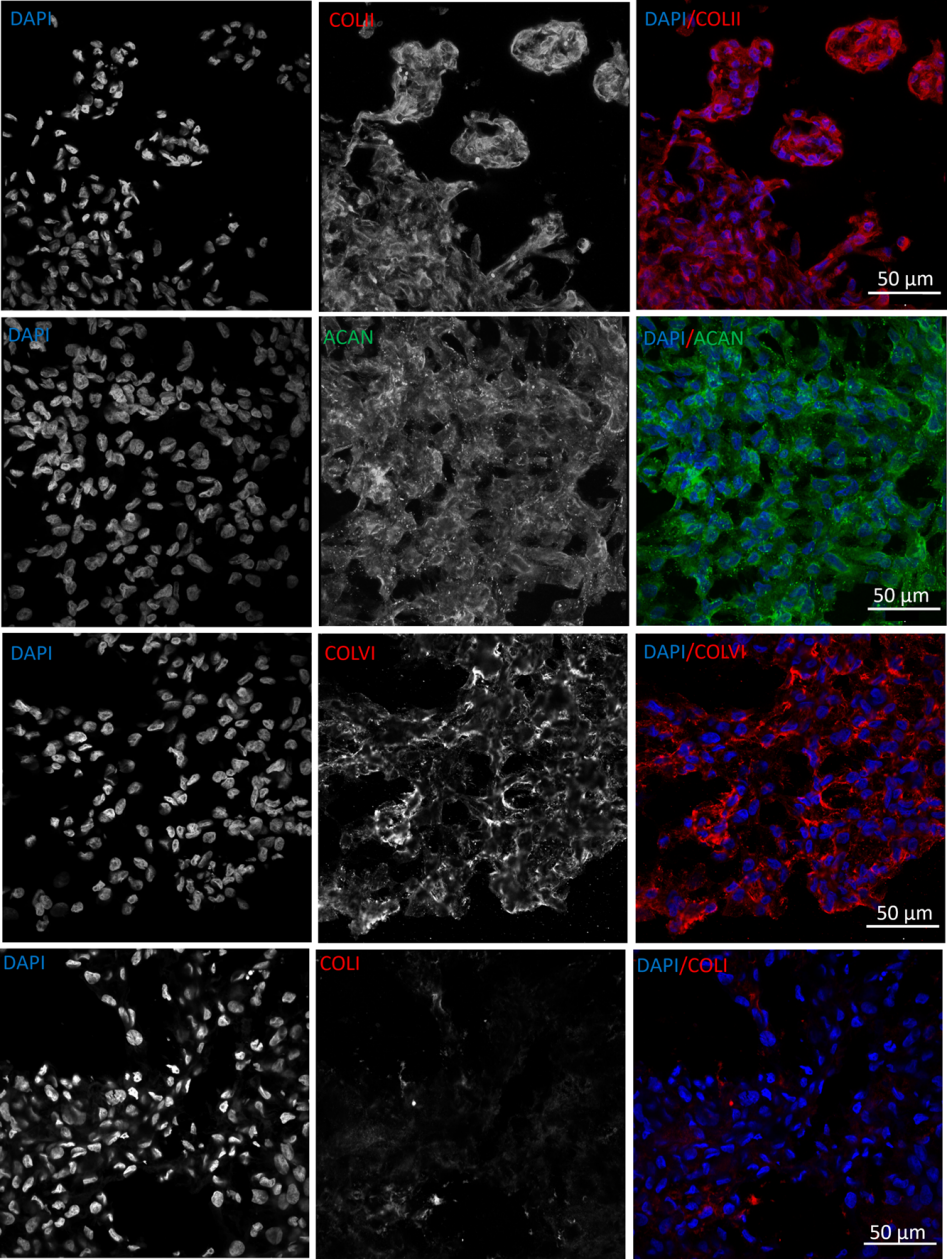
Immunofluorescence images of ECM deposition at day 21. Representative confocal images of P4 chondrocytes embedded into col/alg hydrogels stained for collagen type II, aggrecan, collagen type VI, and collagen type I.

The ability of chondrocytes to deposit cartilaginous ECM when embedded into col/alg hydrogels without any growth factor was evaluated at day 21 by immunofluorescence staining of collagen type II, aggrecan, and collagen type VI as cartilaginous markers, and collagen type I as a hypertrophic marker. The results demonstrated that when embedded in col/alg hydrogels, P4 chondrocytes regained their capacity to deposit matrix components, as indicated by positive immunofluorescence staining for aggrecan, collagen type II, and collagen type VI (Figure 6). This suggests that the chondrocytes were able to produce and secrete cartilaginous ECM proteins within the hydrogel environment. Furthermore, on day 21, chondrocytes aggregated within the hydrogels began to exhibit tissue-like characteristics, indicating their progressive maturation and organization. Importantly, immunostaining analysis revealed no evidence of collagen type I deposition after 21 days, suggesting that the chondrocytes maintained a nonhypertrophic phenotype.

## 4 Discussion

Data presented in this work demonstrated that chondrocytes grown on a flat tissue culture plastic develop a hypertrophic phenotype. However, when the hypertrophic cells are transferred to the 3D environment of soft col/alg hydrogels, they become spherical and aggregate into clusters; moreover, the production of extracellular matrix proteins occurs. These characteristics indicate that when dedifferentiated chondrocytes are grown in the soft col/alg hydrogel, they redifferentiate into chondrocytes and regain the ability to deposit ECM composed of collagen II and aggrecan. Importantly, the change in the phenotype of these cells is accomplished without using growth factors. The main drawback in current cell-based therapies is the dedifferentiation of chondrocytes.^6,37^ The loss of chondrocytes' original phenotype leads to the production of fibrocartilage, which has inferior mechanical properties compared to hyaline cartilage.^30^One of the main strategies to re-establish chondrocytes original phenotype is the use of growth factors, such as TGF-β, moreover, growth factors could promote the expansion of chondrocytes obtained from elderly patients.^38−41^ However, the use of growth factors raises the issue of their clinical biosafety. As they induce rapid proliferation of cells that may favor damage accumulation in DNA, uncontrolled proliferation, and genomic instability; thus increasing the risk of tumorigenic transformation.^43−45^ Furthermore, the use of TGF-β for redifferentiating chondrocytes has been shown to promote the expression of hypertrophic markers, which will eventually lead to hypertrophy and cartilage mineralization.^15,17,46^

Finding strategies to reprogram chondrocytes without the use of growth factors and preventing hypertrophy is vital in order to repair and regenerate cartilage tissue. We previously demonstrated that soft col/alg hydrogels supported the differentiation of oMSCsAQ and limited the deposition of collagen type I.^41^ The effect of soft col/alg hydrogels on P4 chondrocytes was investigated in order to evaluate whether they can support the reprogramming of chondrocytes to their original phenotype without the use of growth factors. Chondrocytes were characterized at P1 and P4 and as expected, when expanded in 2D, they lost their original phenotype, acquiring a spindle-shape morphology, and lost the ability to deposit ECM at P4. At P4 chondrocytes were embedded into col/alg hydrogels, and their response in terms of viability, proliferation, gene expression, and ECM deposition were evaluated. The results reported in this work showed that chondrocytes were viable and proliferated within the hydrogel, and chondrocytes regained their original morphology and formed aggregates after only 7 days of culture. In accordance with other studies, dedifferentiated chondrocytes started re-expressing chondrogenic markers upon the restoration of a spheroidal shape.^6,47−52^ In this study, after 7 days of culture it was possible to observe an increase in the expression of FOXO3A, COL6A1, and ACAN, while the expression of COL1A1 and COL10A1 decreased. On day 7, chondrocytes started to self-assemble, suggesting that they were able to migrate within the hydrogel. It is well-known that chondrocyte aggregates promote cell differentiation and ECM synthesis by mimicking the in vivo environment, which allows cell-to-cell contact and cell-to-ECM contact.^44^ The use of spheroids to repair cartilage defects in the knee is in clinical use where spheroids measuring 500–800 μm in diameter are generated from autologous chondrocytes and injected in vivo*.*^53^ While spheroids have been used in clinical settings for repairing cartilage defects, they can present challenges such as the development of a necrotic core due to limited nutrient diffusion and difficulties in handling during surgery.^54^ Additionally, the production of spheroids and their subsequent encapsulation into hydrogels can be time-consuming and costly. Conversely, in our study, P4 chondrocytes in col/alg hydrogels were able to migrate, self-assemble, and form aggregates in just 7 days of culture. Furthermore, an important observation was that the chondrocyte aggregates formed within the col/alg hydrogels did not exhibit any signs of a necrotic core, as confirmed by the live/dead staining. This is a significant finding, as it indicates that the aggregates remained viable and metabolically active throughout the culture period.

The match between tissue deposition and hydrogel degradation rate is a critical consideration in the design of tissue engineering scaffolds. In an ideal scenario, the rate of tissue deposition should be balanced with the rate of hydrogel degradation to ensure the long-term stability and functionality of the scaffold. If tissue deposition outpaces hydrogel degradation, then the scaffold may become insufficient to support the growing tissue, leading to mechanical instability and potential failure. Conversely, if hydrogel degradation outpaces tissue deposition, the scaffold may collapse prematurely, compromising the structural integrity and inhibiting proper tissue formation.^36,55,56^ In the context of our study, we observed that chondrocytes aggregated and were able to support ECM synthesis by day 21, suggesting that tissue deposition occurred within the time frame of hydrogel degradation. This suggests a level of coordination between tissue formation and hydrogel degradation, allowing for the maintenance of a coherent structure until new tissue forms.^41^ This demonstrates the potential of col/alg hydrogels to create a microenvironment that promotes cell–cell interactions and ECM deposition, leading to the in vivo development of functional cartilaginous tissue. One of the most remarkable findings in this study was the notable absence of collagen type I expression on day 21, which strongly indicates that the utilization of col/alg hydrogels facilitated the redifferentiation of chondrocytes and effectively prevented chondrocyte hypertrophy and the formation of fibrocartilage. This result underscores the potential of col/alg hydrogels as a promising approach to promote chondrocyte reprogramming and facilitate the regeneration of cartilaginous tissue. Our study stands out for its focus on reprogramming the chondrocyte phenotype within a three-dimensional context, achieving this feat without the use of growth factors. Growth factors present challenges in clinical translation due to potential biosafety concerns and their tendency to induce hypertrophy marker expression. This necessitates the exploration of alternative strategies. Chondrocytes are highly sensitive to the mechanical cues of their surrounding ECM within the physiological environment. ECM stiffness plays a pivotal role in dictating chondrocyte behavior, a phenomenon observed in both traditional monolayer cultures and 3D cultures. The stiffness of the chondrocyte-biomaterial interface profoundly influences various cellular behaviors including cell morphology, functionality, proliferation, and migration. While previous research has explored the ability of soft substrates in promoting a more chondrogenic phenotype in 2D, the effect of 3D culture environments on chondrocyte redifferentiation remains largely underexplored.^55,57,58^ We have previously highlighted the critical role of soft hydrogels in emulating the mechanical milieu of developing cartilage, which may significantly influence cell fate compared to scaffolds mimicking mature cartilage properties.^41^ In our current study, we provide further evidence supporting the advantageous use of soft hydrogels in promoting chondrogenesis and facilitating chondrocyte redifferentiation within a 3D setting, a feat not previously achieved in a growth factor-free manner. This underscores the importance of meticulously investigating the mechanobiology of scaffolds, as they offer a promising avenue for manipulating the cellular phenotype through the modulation of the biomaterial–cell interface.

It is worth noting that the dedifferentiation process of chondrocytes from P1 to P4 in this study occurred over a period of 4 to 6 weeks, which aligns with the current estimated time required for the growth of articular chondrocytes in autologous chondrocyte implantation (ACI) procedures. To further explore the potential of soft col/alg hydrogels, additional ex vivo and in vivo studies should be conducted. These studies would allow for the evaluation of the long-term deposition of the extracellular matrix and the integration of the generated tissue with the native cartilage. Furthermore, investigating the effect of col/alg hydrogels on maintaining chondrocyte phenotype could be considered as an alternative approach to traditional 2D culture methods for the in vitro expansion of chondrocytes. One of the limitations of this study was the inability to identify relevant controls, which led us to make a comparison only between cells before and after encapsulation into the hydrogels. Using pellets as controls was not considered suitable, as our primary objective was to analyze the isolated effect of hydrogels on chondrocytes without the influence of any growth factors. Maintaining pellets in culture usually requires the presence of growth factors, which could introduce confounding variables and affect the intended isolation of hydrogel effects.^59−61^ Furthermore, pellets typically require a higher cell count, around 200,000 cells per pellet, which differs from the cell count used in our experiments.^60−62^ Moreover, another important aspect to consider when using pellets is cell-to-cell interactions. During pellet culture, cells are compacted and in close proximity to each other. It is well-known that cell-to-cell interactions can influence gene expression and matrix deposition.^63−67^ This suggests that pellet culture does not fully replicate our model, in which cells within col/alg hydrogels are seeded as single cells and form aggregates only after 7 days of culture. To ensure the accuracy and integrity of our results, we opted to avoid using pellets as controls and instead designed our experiments to directly assess the impact of hydrogels on chondrocytes without the interference of external factors. The main limitation in the use of soft col/alg hydrogels in the repair of AC is that this type of hydrogel will not be able to withstand the loads to which cartilage is subject to. A preculture of chondrocytes will be required before implantation in order to allow the formation of ECM, which would be expected to increase Young’s modulus of the hydrogel–chondrocyte combination. A possible solution would be to increase the mechanical properties of the col/alg hydrogels; however, this may affect the stiffness of the gel and alter the phenotypic behavior of the cells.

## 5 Conclusions

In conclusion, our study demonstrates that chondrocytes cultured on flat tissue culture plastic tend to develop a hypertrophic phenotype, presenting a significant challenge for cartilage repair strategies. However, when these hypertrophic cells are transferred to the 3D environment provided by soft collagen and alginate (col/alg) hydrogels, they undergo remarkable phenotypic transformation. Within the hydrogels, chondrocytes regained their spherical morphology, formed clusters, and initiated production of key ECM components, such as collagen II and aggrecan. Importantly, this redifferentiation occurred without exogenous growth factors, eliminating concerns about biosafety and potential hypertrophy induction. These findings suggest that col/alg hydrogels may promote chondrocyte reprogramming and facilitate cartilage regeneration. The limited collagen I deposition on day 21 indicates the hydrogel’s effectiveness in preventing hypertrophy and fibrocartilage formation. This underscores the importance of a biomimetic microenvironment that replicates the mechanical cues of native cartilage for guiding the cell fate. These findings highlight the potential of col/alg hydrogels as a promising approach for cartilage repair. The biomimetic properties of the hydrogels may offer advantages over current methods by promoting redifferentiation and preventing hypertrophy in a growth factor-free environment.

## Data Availability

The data presented in this study are available at DOI: https://doi.org/10.17029/bc22ffc4-63f3-43be-84fa-a6b32bd059f0.

## Supporting Information Available

Validation of CFSE staining protocol on chondrocytes in 2D; viability of chondrocytes stained with CellTrace CFSE at 5 and 10 μM at different time points; and flow cytometry analysis of CFSE labeling efficacy of oMSC at different concentrations and time points (PDF)
