# Supplementary material for: Collagen and Alginate Hydrogels Support Chondrocytes Redifferentiation In Vitro without Supplementation of Exogenous Growth Factors
Source: ACS Omega. 2024 May 4;9(19):21388–400. doi: 10.1021/acsomega.4c01675 (PMC11097186; doi:10.1021/acsomega.4c01675)
Supplement: Supplementary file 1 — ao4c01675_si_001.pdf [file ao4c01675_si_001.pdf]

Collagen and alginate hydrogels support chondrocytes re-differentiation in vitro without supplementation of exogenous growth factors

*Tosca Roncada[1a], Gordon Blunn[1], Marta Roldo[1] **

[1] University of Portsmouth, School of Pharmacy and Biomedical Sciences, St Michael's Building, White Swan Road, Portsmouth, PO1 2DT, UK.

[a] Current address: Trinity Centre for Biomedical Engineering, Trinity Biomedical Sciences Institute, Trinity College, 152-160 Pearse Street, Dublin 2, Ireland. D02 R590

* Correspondence: marta.roldo@port.ac.uk

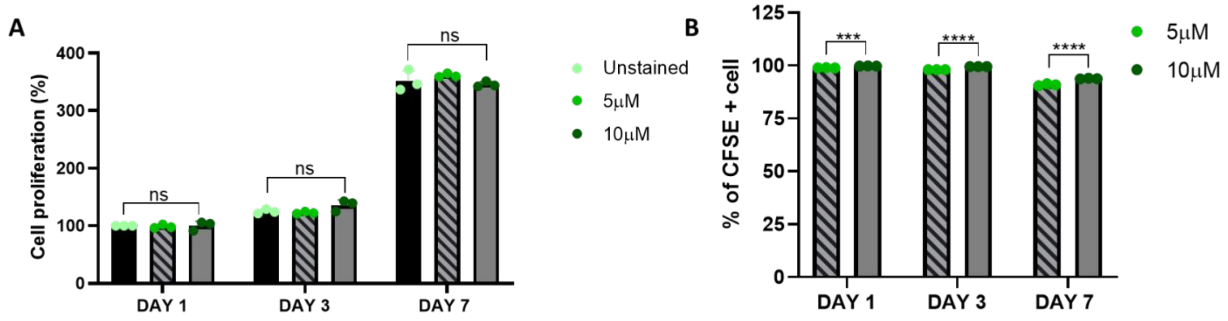

Figure S1. Validation of CFSE staining protocol on chondrocytes in 2D. (A) Viability of chondrocytes stained with CellTrace CFSE at 5 and 10 μM at different time points. Error bars denote standard deviation, n = 3. Comparison between groups was assessed by ordinary two-way ANOVA using post hoc Tukey's test, (B) Flow cytometry analysis of CFSE labelling efficacy of oMSC at different concentrations and time points. Error bars denote standard deviation, n = 3. Comparison between groups was assessed by ordinary two-way ANOVA using post hoc Sidaks's test, *** $p = 0.0006$, **** $p < 0.0001$
